# Supplementary material for: On the modelling and testing of a laboratory-scale Foucault pendulum as a precursor for the design of a high-performance measurement instrument
Source: Proc Math Phys Eng Sci. 2020 Jun 3;476(2238):20190680. doi: 10.1098/rspa.2019.0680 (PMC7428043; doi:10.1098/rspa.2019.0680)
Supplement: ESM1_Derivation of the equations of motion [file rspa20190680supp1.pdf]

ESM file 1: *On the modelling and testing of a laboratory scale Foucault pendulum as a precursor for the design of a high performance measurement instrument*, by M.P.Cartmell, J.E.Faller, N.A.Lockerbie & E.Handous

Derivation of the equations of motion

It is easily seen from Figure 1 that the global and local unit vectors associated with the Earth's rotation can be expressed by,

$$\bar{e}_Z = sin\phi.\bar{e}_z + cos\phi.\bar{e}_y \tag{S1}$$

Therefore,

$$\bar{\Omega} = \begin{pmatrix} 0 \\ 0 \\ \Omega \end{pmatrix}_{(XYZ)} = \begin{pmatrix} 0 \\ \Omega\ cos\phi \\ \Omega\ sin\phi \end{pmatrix}_{(xyz)} \equiv \begin{pmatrix} \Omega\ sin\phi \\ 0 \\ \Omega\ cos\phi \end{pmatrix}_{(zxy)} \tag{S2}$$

The position vector from the origin of the global frame to the pendulum bob B is given by $\overline{EB}$ from Figure 1, and if we project a horizontal line back to the pz axis in Figure 2 to the point C, then by using Chasles' relation we see from Figures 1 and 2 that the following applies,

$$\overline{EB} = (\overline{EC}) + (\overline{CB}) = (r\bar{e}_z) + (x\bar{e}_x + y\bar{e}_y) \tag{S3}$$

where $r = r_E + \|\overline{pC}\|$, noting that r_E is the radius of the Earth to the grounded origin p of the local frame, and that $\|\overline{pC}\|$ ($\equiv AB$) is the height of the pendulum pivot above the ground, otherwise denoted by h. The velocity of the pendulum bob, M, measured with respect to the global frame's origin, is given by the first time derivative of equation (S3), hence,

$$\frac{d\overline{EB}}{dt} = \frac{d(r\bar{e}_z + x\bar{e}_x + y\bar{e}_y)}{dt} \tag{S4}$$

Expanding the derivative in full leads to,

$$\frac{d\overline{EB}}{dt} = \dot{r}\bar{e}_z + r\frac{d\bar{e}_z}{dt} + \dot{x}\bar{e}_x + x\frac{d\bar{e}_x}{dt} + \dot{y}\bar{e}_y + y\frac{d\bar{e}_y}{dt} \tag{S5}$$

We note here that the derivatives of the unit vectors in equation (S5) are with respect to the global frame, and so we need to invoke the transformation of equation (S2) to obtain the following results for the derivatives of the unit vectors of the local frame, doing the remaining arithmetic in (zxy) to be consistent with the order of terms taken in equations (S3) to (S5),

$$\frac{d\bar{e}_z}{dt} = 0 + \begin{pmatrix} \Omega\ sin\phi \\ 0 \\ \Omega\ cos\phi \end{pmatrix} \times \begin{pmatrix} 1 \\ 0 \\ 0 \end{pmatrix} = \begin{pmatrix} 0 \\ \Omega\ cos\phi \\ 0 \end{pmatrix} \tag{S6}$$

$$\frac{d\bar{e}_x}{dt} = 0 + \begin{pmatrix} \Omega\ sin\phi \\ 0 \\ \Omega\ cos\phi \end{pmatrix} \times \begin{pmatrix} 0 \\ 1 \\ 0 \end{pmatrix} = \begin{pmatrix} -\Omega cos\phi \\ 0 \\ \Omega sin\phi \end{pmatrix} \tag{S7}$$

$$\frac{d\bar{e}_y}{dt} = 0 + \begin{pmatrix} \Omega\ sin\phi \\ 0 \\ \Omega\ cos\phi \end{pmatrix} \times \begin{pmatrix} 0 \\ 0 \\ 1 \end{pmatrix} = \begin{pmatrix} 0 \\ -\Omega\ sin\phi \\ 0 \end{pmatrix} \tag{S8}$$

Note that the zeros in the first terms on the right-hand sides of these equations simply account for the fact that the local frame is not physically accelerating.
The height of the pendulum bob above the ground h varies with the swing angle α, therefore $\dot{h} \neq 0$, but $h \ll r_E$ and as the radius of the Earth r_E is considered to be constant at any latitude, then $\dot{r} \approx 0$. We also regard the angular velocity of the Earth Ω and the latitude ϕ as constants.
By using equations (S6)-(S8), the conditions summarised above, and substituting for the numerical components of the unit vectors, we can return to the velocity of the pendulum bob in equation (S5) to get the following,

$$\frac{d\overline{EB}}{dt} = r\begin{pmatrix} 0 \\ \Omega\, cos\phi \\ 0 \end{pmatrix} + \dot{x}\begin{pmatrix} 0 \\ 1 \\ 0 \end{pmatrix} + x\begin{pmatrix} -\Omega cos\phi \\ 0 \\ \Omega sin\phi \end{pmatrix} + \dot{y}\begin{pmatrix} 0 \\ 0 \\ 1 \end{pmatrix} + y\begin{pmatrix} 0 \\ -\Omega\, sin\phi \\ 0 \end{pmatrix} \qquad \text{(S9)}$$

Reinstating the explicit unit vectors for clarity and then expanding equation (S9) leads to,

$$\frac{d\overline{EB}}{dt} = V_M = (\,-x\Omega cos\phi)\bar{e}_z + (\dot{x} + r\Omega cos\phi - y\Omega sin\phi)\bar{e}_x + (x\Omega sin\phi + \dot{y})\bar{e}_y \qquad \text{(S10)}$$

We can now obtain the square of the bob velocity by summing the squares of the constituent parts of the velocity given in equation (S10), as follows,

$$V_M^2 = (\,-x\Omega cos\phi)^2 + (\dot{x} + r\Omega cos\phi - y\Omega sin\phi)^2 + (x\Omega sin\phi + \dot{y})^2 \qquad \text{(S11)}$$

The kinetic energy of the pendulum can be assembled by assuming that the mass of the pendulum M is concentrated within the bob, and is therefore given by,

$$T = \frac{1}{2}MV_M^2 \qquad \text{(S12)}$$

One could easily extend this to include the mass of the pendulum wire and this is discussed further in section 6 in the main body of the paper.

The potential energy of the pendulum is entirely gravitational, and by taking the bob mass contribution we see that reference to Figure 2 leads to,

$$U = Mgl\left[1 - cos\left(arcsin\left(\frac{v}{l}\right)\right)\right] \qquad \text{(S13)}$$

where l is the length of the pendulum from the pivot to the bob, $v = \sqrt{x^2 + y^2}$, and g is the local acceleration due to gravity. Two equations of motion in terms of the generalised coordinates x and y can now easily be derived using computer algebra [21] to undertake two successive applications of Lagrange's equation, as follows,

$$\frac{d}{dt}\frac{\partial T}{\partial \dot{q}_{1,2}} - \frac{\partial T}{\partial q_{1,2}} + \frac{\partial U}{\partial q_{1,2}} = Q_{1,2} \qquad \text{(S14)}$$

where $q_1 \equiv x$ and $q_2 \equiv y$, and the generalised force vector is $Q_{1,2} = \{0 \quad 0\}_{1,2}^T$ for this system where there is no external forcing. Substituting equations (S12) and (S13) into (S14) leads to the following nonlinear ordinary differential equations of motion for the pendulum after some algebraic simplification,

$$\ddot{x} + \eta|\dot{x}|\dot{x} - 2\dot{y}\Omega sin\phi - x\Omega^2 + \frac{gx}{l\sqrt{1-\frac{x^2+y^2}{l^2}}} = 0 \qquad \text{(S15)}$$

$$\ddot{y} + \eta|\dot{y}|\dot{y} + 2\dot{x}\Omega sin\phi - y\Omega^2 sin^2\phi + r\Omega^2 sin\phi cos\phi + \frac{gy}{l\sqrt{1-\frac{x^2+y^2}{l^2}}} = 0 \qquad \text{(S16)}$$

A nonlinear aerodynamic dissipation term is also included in each equation, where the coefficient is defined as,

$$\eta = \rho C_d \pi \frac{R_{bob}^2}{2M} \qquad \text{(S17)}$$

noting that ρ is the density of the air surrounding the pendulum, C_d is the drag coefficient of a cylindrical body (in the case the bob) chosen for turbulent air flow, and R_{bob} is the radius of the cylindrical bob. The two second order nonlinear ordinary differential equations couple strongly with the angular velocity of the Earth, as can be seen in equations (S15) and (S16), through the Coriolis and centrifugal terms.

As the Earth, and therefore the laboratory, rotates at Ω about the polar axis, then the component of this angular rate within the laboratory at latitude ϕ and about local axis pz (Figure 2) will be $\Omega\ sin\phi$. As the pendulum swings through angle α, which is equal to $arcsin\left(\frac{v}{l}\right)$, any transferred angular velocity from the spin of the Earth to the pendulum about its long axis will be $\Omega\ .sin\phi\ .cos\ (arcsin\left(\frac{v}{l}\right))$. The torque associated with this component of angular velocity about the long pendulum axis will therefore be $C_B\ \Omega\ .sin\phi\ .cos\ (arcsin\left(\frac{v}{l}\right))$ where C_B is the torsional coefficient of friction within the pivot, $v = \sqrt{x^2+y^2}$, as stated previously, and $l = l(t)$ as defined in equation (2.3) in the main narrative of the paper. The kinetic energy associated with pure torsional motion of the bob is given by $\frac{1}{2}I_T\dot{\theta}^2$, where I_T is the mass moment of inertia of the bob about the long axis of the pendulum, therefore $I_T = \frac{1}{2}MR_{bob}^2$, and θ is the generalised coordinate associated with torsional motion of the cylindrical bob about the long axis. The additional potential energy will be due to the torsional strain in the wire, and so for reasonably small θ the restoring torque will be defined by $\frac{S_G J}{l}\theta$. In this case S_G is the shear modulus of the pendulum wire (shear modulus is usually denoted by G but that notation is avoided here to remove any confusion with Newton's universal constant of gravitation) and J is the polar moment of area for the wire. For a circular section wire this will be $J = \frac{\pi d_{wire}^4}{32}$ where d_{wire} is the wire diameter. If we also include a damping term proportional to the torsional angular velocity of the wire and bob of the form $C_T\dot{\theta}$ then we can construct an additional equation of motion which captures the principal mechanism for pure torsion in the pendulum, as follows,

$$I_T\ddot{\theta} + C_T\dot{\theta} + \frac{S_G J}{l}\theta = C_B\,\Omega.\,sin\phi.cos\left(arcsin\left(\frac{\sqrt{x^2+y^2}}{l}\right)\right) \qquad \text{(S18)}$$

Numerical values can be calculated or obtained directly for I_T, S_G, and d_{wire}, and l is treated numerically for the parametric excitation as in Figure 3. Appropriate values for the additional system constants and parameters are as follows, C_T = 0.0001 Nms, S_G = 161 * 10^9 Pa (tungsten), d_{wire} = 0.00254 m, C_B = 0.003 Nms, $\theta(0)$ = 0.001 rad, $\dot{\theta}(0)$ = 0 rad/s, all other quantities using the same data as for Figure 3. We can now examine the response in pure torsion predicted by this additional equation of motion. The results for θ with time are found to be generally insensitive to the initial conditions mainly due to the relatively high torsional

stiffness of the 2.54 mm diameter tungsten wire chosen for this first numerical example (noting that we discuss wire material and diameter further in section 4 in the paper). Results are obtained for the damping friction value provided by the manufacturer of the spherical rotating joint, $C_B = 0.003$ Nms. It is this quantity that controls the torque available to drive the pendulum into a torsional response as a consequence of the rotation of the laboratory. The other damping quantity is assumed, from experience, and is given here by $C_T = 0.0001$ Nms.
