# Supplementary material for: On the modelling and testing of a laboratory-scale Foucault pendulum as a precursor for the design of a high-performance measurement instrument
Source: Proc Math Phys Eng Sci. 2020 Jun 3;476(2238):20190680. doi: 10.1098/rspa.2019.0680 (PMC7428043; doi:10.1098/rspa.2019.0680)
Supplement: ESM2_Foucault Pendulum numerical integration [file rspa20190680supp2.pdf]

```
(** ESM2 - 'On the modelling and testing of
   a laboratory scale Foucault pendulum as a precursor for
   the design of a high performance measurement instrument',
by M.P.Cartmell, J.E.Faller, N.A.Lockerbie, and E.Handous **)

ClearAll["Global`*"]
Unset[Once[Print[y[t]; p = y[t]]]]

(** Data for the system constants. Values
 for the acceleration due to gravity, latitude,
and Earth radius are all for Glasgow at 40 m above sea level,
noting that the bob is a cylinder made of tungsten of mass 2 kg
 diameter 2 cm and length 32.73 cm. Pendulum wire tempered steel **)

{l0, l1, g, Ω, ϕ, r, m, ρ, CD, Rbob, tend, CT, SG, CB, dwire} =
  {8, 0.075, 9.8156, 7.2921150 * 10^-5, 0.9750, 6363.18 * 10^3, 2,
   1.189, 1.5, 0.01, 14400, 0.0001, 79 * 10^9, 0.003, 0.000813};

(** Calculation of natural frequency and period **)
```

$\omega_{lin} = \sqrt{g / l_0}$;

```
Quantity[ωlin, "rad/s"]
flin = ωlin / (2 * 3.142);
Quantity[flin, "Hz"]
Tlin = 1 / flin;
Quantity[Tlin, "s"]
f1 = 2 * ωlin;
l = l0 + (l1 * Cos[f1 * t]);

(** Calculation of the aerodynamic damping constant **)

η = (ρ * CD * Pi * (Rbob^2)) / (2 * m);

(** Calculation of the mass moment
 of inertia of the bob along the long axis **)

IT = 0.5 * m * ((Rbob)^2);

(** Equations of Motion for fundamental FP motion **)
```

$$\text{eqn3} = \frac{g\,x[t]}{l\sqrt{1-\frac{x[t]^2+y[t]^2}{l^2}}} - \text{Cos}[\phi]^2\,x[t]\,\Omega^2 - \text{Sin}[\phi]\,\Omega\,(\text{Sin}[\phi]\,x[t]\,\Omega + y'[t]) + (-\text{Sin}[\phi]\,\Omega\,y'[t] + x''[t]) + (\eta * \text{Abs}[x'[t]] * x'[t]);$$

```
eqn4 = g y[t] / (l Sqrt[1 - (x[t]^2+y[t]^2)/l^2]) + Sin[ϕ] Ω (Cos[ϕ] r Ω - Sin[ϕ] y[t] Ω + x′[t]) +
    (Sin[ϕ] Ω x′[t] + y″[t]) + (η * Abs[y′[t]] * y′[t]);

(** Equation of motion for pure torsion about the pendulum wire long axis **)
eqn5 = (I_T * θ″[t]) + (C_T * θ′[t]) + (((S_G * ((π * ((d_wire) ^4)) / (32 * l)))) θ[t]) -
    (C_B * Ω * (Sin[ϕ]) * (Cos[ArcSin[Sqrt[(x[t]^2 + y[t]^2)] / l]]));

(** Numerical integration of the three differential equations **)

system2 = NDSolve[{eqn3 == 0, eqn4 == 0, eqn5 == 0, x[0] == 0.1,
     x'[0] == 0, y[0] == 0.018, y'[0] == 0, θ[0] == 0.001, θ'[0] == 0,
     WhenEvent[x[t] == 0, Once[ Print[y[t]; p = y[t]]]]}, {x, y, θ}, {t, 0, t_end},
    MaxSteps → ∞, AccuracyGoal → 20, PrecisionGoal → 20, WorkingPrecision → 55];

(** Parametric plot of y against x over the chosen integration time,
calculation of the start and end coordinates with tooltips,
and extraction of the y coordinate for x = 0. Red point denotes values at t =
   0 and blue point denotes values at t_end **)

ParametricPlot[Evaluate[{x[t], y[t]} /. system2], {t, 0, t_end}, Epilog →
   {PointSize[0.03], Red, Tooltip[#, #[[1]]] &@Point[{x[0], y[0]} /. system2],
    Blue, Tooltip[#, #[[1]]] &@Point[{x[t_end], y[t_end]} /. system2]}, Frame → True,
  FrameTicks → Automatic, GridLines → Automatic, FrameLabel → {x[t], y[t]}]

(** Time plot evaluations **)

Plot[Evaluate[x[t] /. system2], {t, 0, t_end}, Frame → True,
  FrameTicks → Automatic, GridLines → Automatic, FrameLabel → {time, x[t]}]
Plot[Evaluate[y[t] /. system2], {t, 0, t_end}, Frame → True,
  FrameTicks → Automatic, GridLines → Automatic, FrameLabel → {time, y[t]}]
Plot[Evaluate[θ[t] /. system2], {t, 0, t_end}, Frame → True,
  FrameTicks → Automatic, GridLines → Automatic, FrameLabel → {time, θ[t]}]

(** Listing the start and end coordinates,
extracting the y coordinate of the pendulum for x = 0, calculating Newtonian
 precession over integration time and extrapolated over 24 hours **)

x_0 = x[0] /. system2
y_0 = y[0] /. system2
x_tend = x[t_end] /. system2
y_tend = y[t_end] /. system2
x_cent = 0
y_cent = p
```

```
α_tend = 57.2958 * (ArcTan[Abs[(y_0 - y_cent) / x_0]] + ArcTan[Abs[(y_tend - y_cent) / x_tend]])
α_24 hr = α_tend * (86 400 / t_end)
```
