# Supplementary material for: On the modelling and testing of a laboratory-scale Foucault pendulum as a precursor for the design of a high-performance measurement instrument
Source: Proc Math Phys Eng Sci. 2020 Jun 3;476(2238):20190680. doi: 10.1098/rspa.2019.0680 (PMC7428043; doi:10.1098/rspa.2019.0680)
Supplement: ESM3_Experimental system_Cartmell et al [file rspa20190680supp3.pdf]

ESM file 3: On the modelling and testing of a laboratory scale Foucault pendulum as a precursor for the design of a high performance measurement instrument, by M.P.Cartmell, J.E.Faller, N.A.Lockerbie & E.Handous

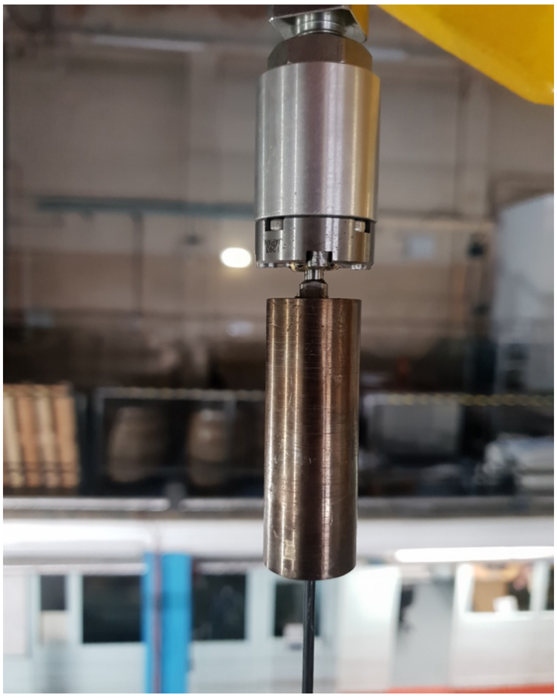

Figure E3.1. Pendulum upper suspension components, showing the linear motor actuator shaft (very top of the picture), the cylindrical adaptor, the spherical rotating joint, and the adaptor connecting this to the pendulum wire.

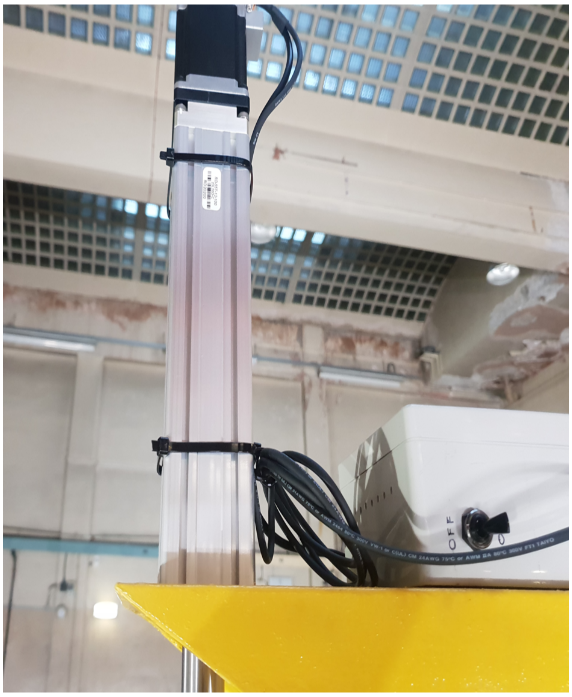

Figure E3.2. Linear actuator for the parametric excitation, fitted to the cantilevered support channel section beam. Note the servo-motor at the top and the housing for the associated drive amplifier electronics to the right-hand side.

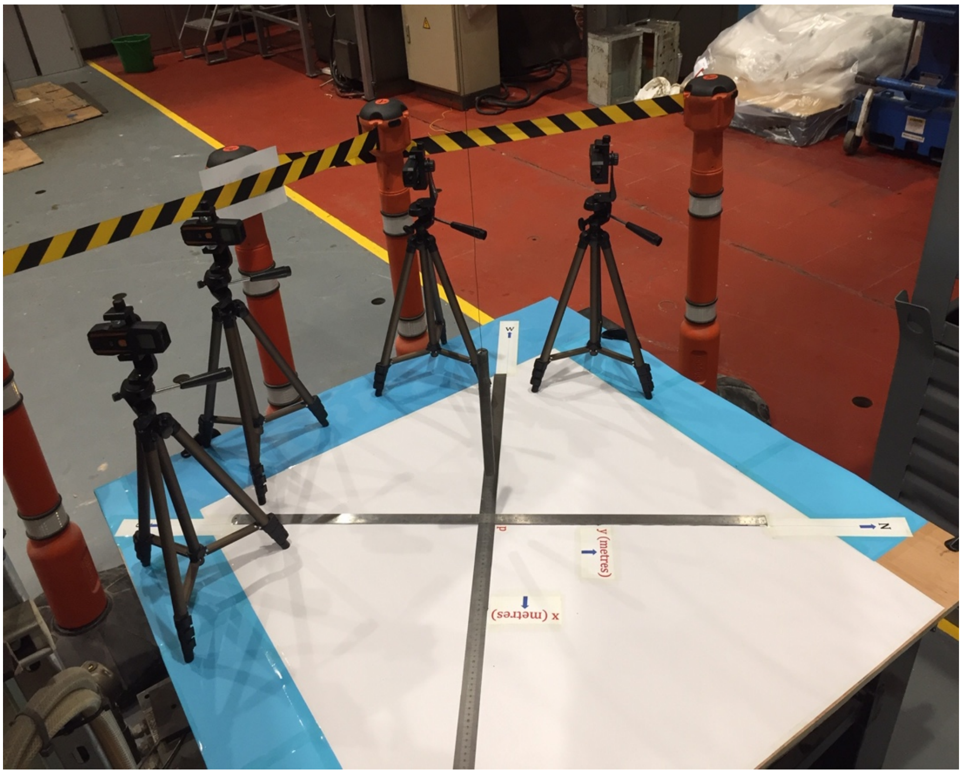

Figure E3.3. Laser rangefinders on the instrumentation table prior to the experimental tests, view taken looking west along the (negative) x axis and with the pendulum shown at rest.
